# Editorial: Exploring the role of exosomes in cancer metastasis

**DOI:** 10.3389/fphar.2025.1721903

**Published:** 2025-12-03

**Authors:** Anil Kumar Kalvala, Venkatesh Pooladanda, Nagavendra Kommineni

**Affiliations:** 1 Department of Biotechnology and Immunotherapeutics, School of Pharmacy, Texas Tech University Health Sciences Center, Abilene, TX, United States; 2 Center for Genomic Medicine, Department of Neurology, Massachusetts General Hospital, Boston, MA, United States; 3 Thermo Fisher Scientific Inc, Cincinati, OH, United States

**Keywords:** extracellular vesicles, integrins, organotropic metastasis, EV biogenesis, EV uptake

Extracellular vesicles (EVs) are nanosized (30–200 nm) lipid bilayer particles that were once regarded as cellular waste products but are now recognized as critical mediators of intercellular communication. Early pioneering work by Rose Johnstone revealed that EVs contain specific protein cargo (Johnstone et al., 1987), and Jan Lotvall later demonstrated that they also carry functional mRNA and microRNA. Subsequent studies identified DNA and other bioactive molecules within EVs, establishing their role in diverse physiological and pathological processes (Lötvall et al., 2014; Malkin and Bratman, 2020). For example, stem cell–derived EVs promote tissue repair (Chen et al., 2017), while cancer-derived EVs facilitate tumor growth and metastasis (Xu et al., 2018). A landmark study by Lyden and colleagues (Nature Medicine, 2012) provided the first evidence that EVs derived from highly metastatic melanoma cells can permanently “educate” bone marrow progenitor cells, thereby enhancing the metastatic potential of primary tumors (Peinado et al., 2012). One of the most intriguing aspects of these vesicles is their ability to promote organotrophic metastasis, the phenomenon where EV cargo dictates the preferred organ destination of metastatic spread. Evidence suggests that this organotropism is largely governed by specific integrin subtypes carried on cancer-derived EVs. The Hoshino et al. (Nature, 2015) study elegantly demonstrated that EVs from tumor cells expressing α6β4 and α6β1 integrins are associated with lung metastasis, while those expressing αvβ5 integrins preferentially promote liver metastasis (Hoshino et al., 2015). Targeting these integrins significantly reduced EV uptake and the corresponding metastatic colonization. Mechanistically, α6β4 and α6β1 bind to laminin, activating c-SRC–S100A4 signaling and promoting lung-tropic metastasis, while αvβ5 binds to fibronectin on liver Kupffer cells, promoting liver-tropic metastasis. Additionally, αvβ3 and αvβ8 integrins in breast cancer EVs facilitate brain metastasis, whereas α4β1 and α5β1 integrins are implicated in bone metastasis (Lu et al., 2020; Yuan et al., 2021). Previous studies have shown that EVs derived from TSC2-null cells can promote the formation of lung pre-metastatic niche in NCG mice. Moreover, plasma-derived EVs from lymphangioleiomyomatosis (LAM) patients exhibited elevated levels of c-SRC, FAK, and other related signaling molecules, consistent with *in vitro* findings (Karbowniczek et al., 2025).

A central question remains: how do these nanosized vesicles so effectively reprogram recipient cells? Do they merely deliver molecular cargo, or do they carry pre-assembled protein complexes capable of instant signaling? Understanding EV topology, the spatial orientation of proteins within the vesicle membrane is crucial to answer this. EV integrins exist as heterodimers, and their cytoplasmic tails often bind to adaptor proteins such as talin and kindlin, which determine the activation state of the integrin receptor (Aretz et al., 2023). These integrin–adhesion complexes enable immediate communication with recipient cells, initiating rapid downstream signaling. Several studies have also demonstrated that cancer-derived EVs contain matrix metalloproteinases (MMP3, MMP7, MMP9, etc.), which enhance metastatic potential either by remodeling the extracellular matrix or by activating integrin signaling pathways (Karbowniczek et al., 2025; Wang et al., 2017; Becker et al., 2016). Furthermore, EVs are enriched in tetraspanins (CD9, CD63, CD81)—classical EV markers whose expression correlates with EV biogenesis. Interestingly, tetraspanins themselves can contribute to the formation of focal adhesion points, amplifying the migratory and invasive potential of cancer cells (Rana and Zöller, 2012; Ramos, 2022).

Two key determinants govern the oncogenic impact of EVs: how they are secreted and how they are internalized by recipient cells. Although the precise mechanisms of these processes are still being elucidated, several drugs are under investigation for their ability to inhibit EV biogenesis or uptake. Tipifarnib (a farnesyltransferase inhibitor) has been identified through high-throughput screening as a potent inhibitor of both ESCRT-dependent (Alix) and ESCRT-independent (nSMase2) EV biogenesis in metastatic castration-resistant prostate cancer cells (Datta et al., 2018). GW4869, an inhibitor of neutral sphingomyelinase 2 (nSMase2), suppresses EV secretion and has been shown to inhibit proliferation and promote apoptosis in colorectal cancer models (Wang et al., 2019). Cannabidiol (CBD) also reduces EV release and exhibits anticancer potential in various tumor types, though its specific mechanisms remain under investigation (Kosgodage et al., 2018). Dyngo-4A, a dynamin inhibitor, and its analog dynasore block EV uptake by disrupting clathrin-mediated endocytosis, thereby reducing tumor-derived EV internalization and angiogenesis (McCluskey et al., 2013; Fuentes et al., 2020). While these findings highlight the promise of EV-targeted therapies, practical challenges remain mainly in difficulty of selectively targeting tumor-derived EVs without affecting physiological EVs. Regarding EV isolation, current methodologies include ultracentrifugation, ultrafiltration, size-exclusion chromatography, polymer-based precipitation, and affinity-based capture using antibodies have each with its advantages and limitations. According to the MISEV2023 guidelines (ISEV 2023), combining multiple methods is recommended to enhance purity, reproducibility, and yield (Welsh et al., 2024). Before functional assays, it is essential to verify cargo reproducibility and EV integrity across methods used to isolate EVs. Despite significant progress, key limitations persist, including the lack of standardized dose optimization and precise definitions of EV purity. The future of EV research depends on a deeper understanding of EV biogenesis, uptake mechanisms, and protein topology, as these factors determine vesicle function and specificity. In conclusion, effective targeting EVs in cancer requires a comprehensive understanding of their molecular cargo, topology, and signaling mechanisms. Therapeutic strategies that selectively modulate EV communication rather than indiscriminately blocking biogenesis or uptake are more likely to yield safe and successful outcomes. Looking forward, microfluidic chip technologies hold promises for rapid, noninvasive tumor detection from a single drop of patient blood, marking an exciting frontier in cancer diagnostics and precision medicine using EVs.
